# Acute and transient psychosis: A paradigmatic approach

**DOI:** 10.4103/0019-5545.37662

**Published:** 2007

**Authors:** Savita Malhotra

**Affiliations:** Prof. of Psychiatry, Post Graduate Institute of Medical Education and Research, Chandigarh - 160012, India

Dr. Reddy, President IPS and Chairman of this session, all my friends and colleagues in the IPS, ladies and gentlemen, it is indeed a great privilege and pleasure for me to be chosen for the DLN Murthy Rao Oration Award for 2007. I am highly thankful to the IPS for this honour which is also its highest award. The topic for the oration “Acute and Transient Psychosis: A Paradigmatic Approach” has been selected for two reasons. First, this has been the subject of my research since the 1980s and second, because the subject poses a major challenge to psychiatrists in India and the developing world for insights, as the acute and transient psychosis (ATP), being more common in these countries, lends the best opportunity to them for further research.

Research on ATP in India has made a significant contribution and this is one area where Indian research has made a mark at the international level. ATPs provide a paradigm for study of psychoses that are neither schizophrenic nor affective, thus opening newer vistas in the nature and classification of major psychotic disorders known in clinical practice.

Acute and transient psychosis as a descriptive entity was recognized only recently with the advent of ICD-10[1] in 1992, where it is included under psychotic disorder (F23) as a three-digit code. The key features that characterize the disorder are an acute (within 2 weeks) onset in all the cases; presence of typical syndromes which are described as rapidly changing, variable, polymorphic states and typical schizophrenic symptoms; evidence for associated acute stress in a substantial number of cases and complete recovery in most cases within 2-3 months. Apart from these diagnostic criteria, ICD-10[1] also provides diagnostic guidelines which include:

- Not meeting the criteria for manic or depressive episodes although affective symptoms may be prominent.
- Absence of organic causation although perplexity, confusion and inattention may be present.
- Absence of obvious intoxication by drugs or alcohol.

It is evident that the ICD-10[1] intends to clearly differentiate the concept of ATP from those of affective psychoses, organic psychoses and drug-induced psychoses.

It is also mentioned, however, that the “nomenclature of these acute disorders is as uncertain as their nosological status…. “Psychotic disorder” is used as a term of convenience.” The fact remains that systematic clinical information that would guide the classification of acute psychotic states is not yet available” (ICD-10).

Nevertheless, four subtypes are described in ICD-10, which are represented on the fourth digit, i.e.,

F 23.0 Acute polymorphic psychotic disorder without symptoms of schizophrenia.

F 23.1 Acute polymorphic psychotic disorder with symptoms of schizophrenia.

F 23.2 Acute schizophrenia-like psychotic disorder.

F 23.3 Acute predominantly delusional psychotic disorders.

In the end, there are Other (F 23.8) and Unspecified categories (F 23.9) of acute psychoses.

There is provision for coding the presence or absence of stress on the fifth digit. Time limits and transitions from one disorder to another have been included to make *ad hoc* or *post hoc* diagnosis of ATP as the duration criterion is an important factor. Overall, duration of the total episode should not exceed 3 months and that for schizophrenic symptoms should not exceed 1 month. There is provision for change of diagnosis to schizophrenia in case the duration of symptoms exceeds these limits.

## ATP: A NOSOLOGICAL CHALLENGE

ATP is a new entrant to psychiatric nosology and ICD-10 concept of ATP has limited validity. While knowledge is the basis of nosology and classification, nosology facilitates research and further generation of knowledge. Classification is the process by which complexity of phenomena is reduced by arranging them into categories according to some established criteria for one or more purposes. Purpose of classification is to improve treatment and prevention efforts. Ideally, classification of disorders should be based on knowledge of aetiology or pathophysiology. However, in psychiatry, clinical-descriptive approach has been adopted as valid.

When we look at the classification of major mental disorders, we find that it has been based on three major concepts and streams of thought propounded by the three most influential researchers of their time:

Kraeplin (1856-1926) - who classified functional psychoses into dementia precox and manic depressive psychosis on the basis of the course and outcome of the disorders. He believed in organic causation of these conditions.

Bleuler (1857-1939) - gave the term schizophrenia and described the main psychopathological mechanisms underlying the process of schizophrenia, which were mostly inferred in the form of disturbances of associative processes.

Freud (1856-1939) - described neuroses and personality disorders on the basis of psychodynamic theory of personality development arising from unconscious conflicts, dammed-up sexual excitations (actual neurosis) and psychological defence mechanisms.

Between these three schools of thought, most of the known major mental disorders were covered. They used different sets of parameters to describe these disorders. However, in contemporary psychiatry, any psychiatric diagnosis must succeed certain tests of validity given by Feighner *et al.*,[2] i.e., distinct clinical description, delimitation from other disorders, laboratory studies, follow-up studies and family genetic studies, before it can be accepted as a valid diagnostic entity worthy of inclusion in nosology and classification.

Testing the concept of ATP on these tests of validity has been the major nosological challenge. Effort has been made in the paper to examine the available research data for insights and directions into the nature of ATP.

### Historical evidence

There were several reports, from several different parts of the world, of occurrence of certain psychotic states other than schizophrenia and MDP described by different names listed below:

**Table:** 

France:	Bouffee Delirante[3]
Germany:	Motility Psychosis[4]
	Cycloid Psychosis[5,6]
	Reactive Psychosis[7]
Scandinavia:	Psychogenic psychosis[8–11]
	Schizophreniform Psychosis[12]
America:	Remitting Schizophrenia;[13]
	Good Prognosis Schizophrenia[14,15]
	Hysterical Psychosis[16]
	Acute Schizoaffective Psychosis[17]
Japan:	Atypical Psychosis[18]
Africa:	Acute Primitive Psychosis[19]
	Acute Paranoid Psychosis[20]
	Transient Psychosis[21]
West Indies:	Acute Psychotic Reaction[22]
India:	Acute Psychoses of Uncertain Origin[23]
	Hysterical Psychosis[24]
	Acute Psychosis without Antecedent
	Stress[25]
	Acute Schizophrenic Episode[26]

- Common features of these historical entities were:

– acute or sudden onset

– unstable, variable, fluid and florid symptomatology

– volatile polymorphic content

– anxiety

– fear or prominent affective symptoms

– association with a clear precipitant

– good premorbid adjustment

– rapid and complete recovery

- These syndromes did not fit into descriptions of affective or schizophrenic disorders.

Although this evidence was highly suggestive, there was need for confirmation using standardized methodology, i.e., use of standard instruments, reliable assessment procedures and standardized diagnostic criteria, through carefully planned, prospective, systematic studies.

The confirmatory evidence for the validity of ATP came from the international initiatives in the form of WHO multi-centered collaborative studies listed below, directed primarily at the study of schizophrenia (IPSS),[27] first onset psychosis (DOSMeD)[28] and Acute Psychoses (CAP).[29] Sufficient data was generated, which added to the knowledge about ATPs:

1. IPSS (International Pilot-study of schizophrenia (1968-1970):[27] This was a nine-country study on schizophrenia, led and funded by WHO, with the main aims:
  - Whether schizophrenia existed in different parts of the world?
  - What were the common/differing clinical presentations?
  - What was the course and outcome among different cultures?
  This study yielded important data on the occurrence, course and outcome of schizophrenia in different parts of the world. However, certain findings were important for discussion on ATP:
  - That a substantial proportion (26%) of schizophrenic subjects had good outcome in the form of only one episode; and
  - That patients from developing countries had better outcome.
    The question was whether these cases can be considered as good outcome schizophrenias or some other psychosis?
2. DOSMeD (Determinants of Outcome of Severe Mental Health Disorders)[28] (1978-1980): In this study, all the cases of first onset psychosis within a specified catchment area and specified age range were taken. This was again a WHO-led and -funded study done in 10 countries including both the developing and developed countries. This was the first systematic study to provide information on incidence of schizophrenia.
  Salient findings relevant to the discussion on ATP were:
  - Incidence of schizophrenia: Incidence differed for “broadly defined” (including reactive and unspecified psychoses, as per ICD-9) schizophrenias (1.5-4.2/lac/year) and “narrowly defined” (catego S + SFRS) schizophrenias (0.7/lac/year.)
    Thus, the broadly defined schizophrenias did include in its rubric, a group of acute onset reactive and unspecified psychoses.
    There were a group of patients who had non-affective psychosis and which remitted completely. These were called as non-affective, acute, remitting psychosis (NARP). Incidence of such NARP cases was 10 times higher in the developing countries in the DOSMeD data. It is possible that such non-affective, non-schizophrenic remitting psychoses belonged to a different category of psychotic states that were not yet clearly identified in the classificatory systems.
  - These patients from developing countries exhibited a benign course at 2 years follow-up.
  In both the IPSS and DOSMeD, predictors of good outcome were mainly two:
  - Acute onset and
  - Developing country setting
3. CAP (Cross-cultural study of Acute Psychosis) (1980-1982):[29] This study was an off-shoot of DOSMeD study done in 14 centers and 7 countries (Cooper, Jablensky and Sartorius 1990)[23]. Main objectives of this study were to know if there are:
  - Acute psychotic states that can be defined, which are descriptively different from schizophrenia and MDP? and
  - How are acute psychoses related to psychological and physical stress?
  One thousand and four patients meeting the criteria of acute onset of psychotic symptoms, unrelated to any medical, neurological or substance-use condition, were included and data was analyzed. Main findings showed that:
  - A large proportion (41.2%) of acute psychosis patients showed schizophrenic symptoms, whereas 20% showed affective symptoms and 35.3% exhibited other psychoses.
  - 41.7% showed stress close to onset.
  - There was marked prevalence of patients from below-average socio-economic status.
  - 2/3 patients remained well with no relapse at 1 year.
  - Outcome in patients of acute psychosis with schizophrenic symptom was similar to those with only affective symptoms.

Taken together, the findings of these three major WHO studies provided strong evidence in favour of occurrence of acute onset psychotic disorders that were different from both schizophrenia as well as MDP and formed the basis for the ICD-10 category ATP (F23). Although the nomenclature and nosology of acute psychosis was still uncertain, the findings of the above major WHO studies were confirmatory and the evidence was powerful. Thus, ATP came to be recognized as a disorder in ICD-10 (1992).

Sources of uncertainty in the nomenclature and nosology of ATP lied in the issue of the relationship of ATPs with schizophrenia and manic-depressive psychosis. This question became more complicated because ATP had to be defined in reference to schizophrenia and MDP, i.e., with clear separation from both, whereas the boundaries between schizophrenia and MDP itself were not as clear.

The key questions that emerge will be discussed as follows:

- What was the basis for inclusion of ATP in ICD-10?
- What was the basis for its definition and description as was given in the ICD-10?
- Is ATP related to schizophrenia or affective disorders or is it an independent entity?
- What evidence is needed for its further validation?
- Does understanding of ATP help in understanding of the nature of other major psychotic disorders, i.e., schizophrenia, affective disorder?
- Does understanding ATP help in understanding of the psychotic disorders/processes?

## BASIS FOR INCLUSION IN ICD-10 AND ITS DESCRIPTION

It was found that Kraeplinian dichotomy of psychotic disorders did not cover all forms of functional psychoses. There were cases who neither followed the pattern of manic depressive psychosis nor progressed onto dementia-like state. These issues have been amplified by Craddock and Owen[30] in their review paper titled “The beginning for the end for Kraeplinian dichotomy”. There were also emerging descriptions of a “third psychosis” named differently by different workers from various countries of the world, i.e., France, Germany, Scandinavia, America, Japan, Africa, West Indies and India; already listed on page 3 that shared a set of symptoms and clinical descriptions common to all. Results of the three multicentric international WHO studies provided confirmation to the fact that there was evidence for the non-schizophrenia, non-affective remitting psychosis occurring in the setting of stressful events. Acknowledgement of these findings contributed to inclusion of ATP in ICD-10 and also formed the basis for its definitions and description. There were certain unequal key features such as acute onset and polymorphic picture, which were accepted as the defining criteria. Presence of stress was kept as additional feature, which may or may not be present. The exclusionary clauses such as absence of criteria for affective disorders, organic factors, substance misuse, were provided to clearly separate these ATPs from affective disorders, delirium, substance-induced psychiatric syndromes. However, although symptoms of (so-called) schizophrenia could be present in ATP, its separation from schizophrenic disorders was reflected in the duration criteria where the time limits of 1 month for schizophrenic symptoms and 3 months for the total duration of ATP episode were given. Acute psychotic episodes that were beyond these time limits were retrospectively diagnosed as schizophrenias. These specific criteria were at best empirical and needed further research. On the whole, the effort was to separate ATPs from schizophrenias as well as MDPs.

## RELATIONSHIP OF ATPS WITH SCHIZOPHRENIA AND AFFECTIVE DISORDERS

In the post-ICD-10 era, while further studies on ATPs on clinical features, course and outcome were being done, the concepts of schizophrenia and affective disorders were being made trimmer, narrower and homogenous.

Quest to separate ATP from schizophrenia and MDP was complicated as there were questions about the biological distinctiveness of schizophrenias and MDP. There is some consensus about the existence of at least six nuclear syndromes within psychoses, i.e., reality distortion, disorganization, negative, catatonia, mania and depression as summarized by Peralta and Cuesta[31] and authors advocate the necessity for developing diagnostic groupings on such dimensional syndromes.

In a comparative study known as Roscommon Family Study[32–34] of symptoms and outcome of 343 broadly defined schizophrenics and affective illness probands and their 942 first degree relatives (FDRs), it was found that on latent class analysis, six classes emerged:

1. Classic schizophrenia - 26.2%
2. Major Depression - 20.0%
3. Schizophreniform disorder - 18.0%
4. Bipolar schizophrenia - 17.6%
5. Schizodepression - 14.5%
6. Hebephrenia - 3.6%

Notable among these was class 3, schizophreniform disorder seen in 18.0% of patients. According to DSM III R[35] criteria, these patients were diagnosed as non-affective psychosis (54%), schizophrenia (21%) and bipolar illness (15%). As compared to classic schizophrenia, these schizophreniform disorder patients had:

1. Comparable levels of positive psychotic symptoms
2. Relatively lower levels of negative symptoms and deterioration
3. Much shorter episodes
4. More manic symptoms
5. Dramatically better outcome

However, familial risk for schizophrenia and its all non-affective psychoses and all affective illness was similar in classic schizophrenia and schizophreniform disorder. The authors concluded that schizophreniform disorder and schizophrenia are similar disorders. Several studies on schizophreniform disorder, which is a prototypical syndrome of ATP, have yielded varying results. It has been considered to be closely related to affective illness in some studies[11,36,37] and to Schizophrenias in other group of studies[38–40] which reported that schizophreniform disorders are independent psychotic illnesses.

There has been little data and no consensus on whether ATP was related to schizophrenia or to affective disorder. Considering its short duration and complete remission, its closeness to MDP has been favoured by some workers.

While the relationship of ATP to schizophrenia or MDP was under question, the relationship between schizophrenia and affective disorders, the two major psychotic conditions, itself has been a matter of debate. Although most research data supports the distinction between the two disorders to be valid, there have been clinical and research observations to the contrary ever since the historical classification was given by Kraeplin who, in 1920,[41] stated that “It is becoming increasingly clear that we cannot distinguish satisfactorily between these two illnesses and this brings home the suspicion that our formulation of the problem may be incorrect”.

The relationship between schizophrenia and affective disorders has been examined from the standpoint of clinical description, course and outcome and some of the findings that question the dichotomy are included here. Kendell and Gourlay[42–44] tried to discriminate affective disorder from schizophrenia on the basis of various features considered characteristic of each disorder and found a unimodal distribution rather than a bimodal distribution. Brockington[45] reported that the boundary between schizophrenia and affective disorder was blurred. Tsuang *et al.*,[46] in their long-term follow-up studies on change of diagnosis, reported that diagnosis of early affective disorder changed to schizophrenia in 10% and diagnosis of early schizophrenia changed to affective disorder in 5% cases. These descriptive studies also pointed towards the possibility that there may be some degree of overlap between schizophrenia and affective disorders as diagnosed by the current criteria and classificatory systems.

Thereafter, several researchers questioned the Kraeplinian dichotomy on the basis of family and twin studies, as these two disorders failed the tests of validity.

Some of the studies, as cited below, reported that there was a higher risk for affective disorder among relatives of schizophrenic subjects and vice versa.

- Rudin[47] reported that families of schizophrenic probands had equal number of relatives with schizophrenia and affective illness (about 6.2%); and higher number of relatives with non-schizophrenic psychosis (10.3%).
- Powell *et al.*[48] reported that Scottish parents with MDP had both MDP and schizophrenic children in the ratio of 3:2.
- Abrams and Taylor[49] found that there was an excess of affective illness (6.9%) in the FDRs of schizophrenia probands.
- Frangos *et al.*[50] and Baron *et al.*[51] reported that there was a higher risk for affective disorder among relatives of schizophrenia patients.
- Angst *et al.*[52] reported that morbid risk for schizophrenia (1.9%) and schizoaffective disorder (1.5%) was higher than expected among the relatives of MDP.
- Similarly, Scharfetter and Nusperl[53] found that morbid risk for schizophrenia among relatives of affective disorder probands was higher (3.3%) than expected (0.6%).
- Tsuang *et al.*[54] found that risk for bipolar affective disorder was similar among relatives of schizophrenia and bipolar probands.

In summary, it was seen that the risk for affective disorders among FDRs of schizophrenics was 6-8%; and the risk for schizophrenia among relatives of affective disorders was 0.5-3.5% and both these risks were much higher than the risk in the general population for the respective disorders. Genetic overlap was greater when affective illness patients had psychotic symptoms.[55,56] Thus, for genetic studies on affective illness, affective symptoms and psychotic symptoms were examined separately as well.

Clinical research in the last 50 years[57] and genetic research[58] have sufficiently shown that there is no gap between the two prototypes, schizophrenia and mood disorders, but bridges and overlaps.

It was proposed by some workers, that genetically, schizophrenia and affective disorders could lie on a continuum. Earliest evidence of shared liability among subgroups of patients with schizophrenia and affective illness was reported by Slater and Schields,[59] Rosenthal and Kety,[60] which was further supported by Farmer *et al*.[61] Tsuang *et al.*[55] concluded that “it may be possible that some affective disorders with psychotic symptoms could be genetic variants of schizophrenia. Several other groups, working on the genetics of these disorders, reported findings consistent with the expression of liability along a continuum from affective disorder through schizoaffective disorder to schizophrenia depending upon the degree of liability and the exposure to various neurologically damaging factors.

There were further questions on the nature of this shared genetic liability in schizophrenia and affective illness. Could the shared genotype be for propensity, for psychosis and the environmental factors should largely determine whether the genetically vulnerable individual would be unaffected or develop affective illness or schizoaffective disorders or schizophrenia. Thus emerged the continuum hypothesis for schizophrenia and affective disorders. Winokur,[62] Crow[63] and Maier[64] suggested that there was schizophrenia- affective disorder continuum. The question arises, where does acute and transient psychosis figure on this shared genetic liability hypothesis? There are not many studies on this issue.

Family Genetic Studies of ATP: In a major case control study comparing the history of schizophrenia, affective disorder and ATP in the first degree relatives of 40 schizophrenic and 40 ATP patients, it was found that family history of ATP was three times greater and that of schizophrenia was four times lower in the FDRs of ATP as compared to the FDRs of schizophrenic subjects.[65] Further in this study, history of schizophrenia was seen in FDRs of those ATP patients who had schizophrenic symptoms. Family history of affective disorder was similar in FDRs of ATP and schizophrenic probands in this study. These findings gave evidence that ATP is genetically distinct from MDP and that there is genetic overlap between ATP and schizophrenia and schizophrenic symptoms. Genetic distinctiveness of ATP was found in earlier studies on FDRs of atypical psychosis where it was reported that they exhibited more of acute psychosis than bipolar affective disorder or schizophrenia.[66,67]

Das, Malhotra and Basu[68] studied the association of stress as the fifth-digit code in ATP patients diagnosed as per ICD-10 and genetic vulnerability factor in ATP and schizophrenia probands. ATP patients with higher stress experience preceding the onset had lower genetic liability and vice versa. Support for evidence of stress-vulnerability hypothesis for ATP was thus found in this study.

It became amply clear from the above that not only Kraeplin's dichotomous approach to classification of functional psychoses into schizophrenia and MDP was blurred on several counts, there possibly existed “a third” form of functional psychosis evidence which could be summarized below:

- Schizophrenia-MDP dichotomy did not over the entire range of functional psychoses known. At least 20% cases who did not fit either of the descriptions were left undiagnosed.
- 10-30% of functional psychoses presented as intermediate forms, e.g., schizoaffective disorder exhibiting features of both schizophrenia as well as affective disorders.
- Descriptive studies failed to provide a clear separation of psychoses into well-defined clusters by classical symptoms approach.
- There was definite evidence in favour of shared genetic liability between the two disorders, i.e., schizophrenia and MDP.

All this evidence paved the way for further studies on ATP or possible “third” form of functional psychosis.

## ATP: VALIDATION STUDIES

Chandigarh Acute Psychosis Study: This was a major study of acute onset psychosis cases where acute onset psychosis, defined by specific criteria were studied[69] and the findings at the Chandigarh centre revealed that only 60% cases of acute psychosis fitted the criteria for diagnosis of schizophrenia and MDP as per ICD-9 (version used then) and catego. The remaining 40% cases were of non-schizophrenia, non-affective psychosis and could be considered as “ACUTE PSYCHOSES”. These acute psychoses cases had greater frequency of stress (28/42) as compared to that in schizophrenia and MDP cases (9/62) and presented with undifferentiated symptomatology fitting the description of acute polymorphic psychoses.[69] Factor analysis of symptoms in this study yielded six factor structures listed below.

### Chandigarh CAP study

Classification of acute psychoses using factor analysis yielded six factor structures, namely: [Table 1]

**Table 1:** Classification of acute psychoses using factor analysis yielded six factor structures, namely

	Significant concordance with	
	ICD-9	Catego
MDP	MDP	MDP
Confusional psychosis	-	-
Schiozophrenic psychosis	Schizo	S.P.
Reactive hysteriform psychosis	Non org. psy.	-
Reactive undifferentiated psychosis	-	S.P.
Hallucinatory syndrome	-	-

- Factors 1 and 3 were typical syndromes and all others atypical.
- Large number of acute psychosis cases had no corresponding diagnosis in ICD-9 on catego.

Corresponding concordance of factor structures revealed that there was high concordance between factor I (named as MDP) with ICD-9 and catego diagnosis of MDP; and factor III named as schizophrenic psychosis with ICD-9 diagnosis of schizophrenia and catego categories of S and P. Factor IV named as Reactive Hysteriform psychosis had a counterpart in ICD-9 as non-organic psychosis; and had concordance with catego S and P and no concordance with any diagnosis in ICD-9.[70] In this study, only factors I and III were typical syndromes of schizophrenia and MDP, for other factors that appear to be domains of psychopathology cutting across diagnostic boundaries, there were no corresponding diagnostic categories in ICD-9 or catego, thus pointing to the limitations of the classificatory systems to provide diagnosis for a large chunk (40%) of acute psychosis cases.

## ICMR ACUTE PSYCHOSIS STUDY[71]

Study of phenomenology and natural history of acute psychosis was done at four centers in India (Bikaner, Goad, Patiala, Vellore) as ICMR collaborative study. Date on 323 cases of acute psychosis (as per ICD-9) that contributed 9% of total psychosis cases presenting at these sites was reported. It was found that 35% of cases were categorized as Schizophrenics, 25% as MDP and 40% as non-organic psychosis as per ICD-9; and 52% of cases of acute psychosis could not be categorized into any of the catego diagnosis. These findings were on the same lines as the CAP study findings.

### Further analysis of DOSMeD data

In this study of first-onset psychosis cases, it was found that acute psychosis was more prevalent in developing countries and among females.[72] These authors identified these cases as non-affective, acute remitting psychoses (NARP). Duration of episode among non-affective acute psychoses had a bimodal distribution with a point of rarity between the cluster of symptoms where 80% patients had a duration less than 28 weeks and 20% had a duration of more than 1 year.[73,74] Further, 14 out of 17 patients of acute remitting psychosis, maintained full recovery throughout 12 years of follow-up.[75] Considering the duration of episode; acute remitting psychosis had a modal distribution of 2-4 months[76] which is larger than 1-3 months given in ICD-10 for ATP. Findings of these studies provide data for typical duration of ATP episodes which is upto 28 weeks and that is definitely longer than was recognized in the ICD-10.

### Chandigarh ATP course and outcome studies

In a short-term (5 years) follow-up study of ICD-10 ATP cases, it was found that majority (75%) had good outcome in the form of complete recovery and no residual symptoms. Female gender, presence of stress at onset and absence of schizophrenic symptoms predicted good outcome.[77] In a long-term 20-year follow-up study of WHO CAP study cohort Malhotra 2000,[78] up to 82% patients had an excellent outcome with no relapse and no residual symptoms.

Diagnostic stability on follow-up studies of ATP cases was reported to be high. Jorgensen[79] reported that diagnosis was stable in 87% cases of ATP over 8-year follow-up period. At 1-year follow-up, diagnostic change was seen from ATP to schizophrenia in 15% and from ATP to affective disorder in 28% cases.[80] In a 12-year follow-up study of acute psychosis from Chandigarh DOSMeD cohort, 1 out of 17 cases developed schizophrenia and none developed affective disorder.[75]

### Recurrence in ATP

Recurrence was examined in ATP follow-up studies and varying rates have been reported. Malhotra *et al.*[81] reported a recurrence rate of 46.6% on 8-year follow-up; whereas Rozario *et al.*[77] found recurrence in 35% cases of ATP on 5-year follow-up.

Recurrence in the DOSMeD cohort of ATP cases was 22% at 5 years[73,74] and 11.76% at 12-year follow-up.[75] In the study by Malhotra *et al.*,[81] recurrent and non-recurrent ATP cases did not differ on any of the antecedent variables; and recurrent episodes showed consistency in the clinical picture of ATP. Recurrent episodes did not show any seasonal pattern.

### Antecedent factors in ATP

Most of the studies done at Chandigarh reported major findings in socio-demographic factors and significant stressful events associated with the onset of ATP:

- Female preponderance: ATP is reported to be occurring more commonly among females.[73,74,82] This finding was also supported by Pillman *et al*.[83]
- Low socio-economic status and rural population: ATP cases are reported to be more frequently belonging to lower socio-economic status and rural areas.[73,74,82]
- Stress preceding the onset was seen in about 60% of ATP patients and stress was more common among female subjects and was associated with better outcome.[84]
- Types of psychosocial stress experienced by males and females in ATP were different.[85]
- There was history of childbirth within 3 months prior to onset among female patients of ATP[82] as compared to acute onset schizophrenias or affective disorder.
- History of non-specific, short-lasting fever without any associated clinical or lab finding, preceding the onset of ATP was found more frequently among ATP patients as compared to acute onset schizophrenia and affective disorders cases.[82]
- Seasonal pattern was examined for onset of ATP patients and it was found that onset of ATP had a summer peak as compared to schizophrenia patients.[82]

- There is no such comparative data available from other studies except for the report of ICMR acute psychosis study, where psychological stress was seen in 50% of all cases and physiological or somatic stress, mostly febrile illness or childbirth was seen in another 20% of cases. Male female distribution was 60:40 in this study. Few studies that are available have proposed somewhat similar ideas. Acute schizophrenic episode could be considered to be a culture-related syndrome,[86] whereas Fisch[87] described psychosis precipitated by marriage as a culture-bound syndrome. Acute Psychotic manifestations in patients suffering from organic brain infections have been reported by Frasca *et al.*,[88] Jarvis *et al.*,[89] Klein *et al.*[90] and Srikanth *et al*.[91]

## HYPOTHESIS ABOUT THE NATURE OF ATP

All the descriptive data provided a fair picture of ATP and the research findings could be summarized as below:

- ATP is a descriptively valid entity on the basis of onset, duration, course and outcome. ATP presents with cross-sectionally prominent psychotic, affective, confusional symptoms. Diagnostic criteria particularly duration of episode given in ICD-10 is short and needs to be changed to 6 months at least.
- There is suggestive evidence of genetic distinctiveness of ATP.
- Schizophrenia symptoms in ATP and in schizophrenia appear to have shared genetic liability.
- Environmental factors such as fever, childbirth, seasonality, low SES, stress, rural living, seem to be involved in triggering ATP.
- Course and outcome of ATP is different from that of schizophrenia or of affective disorder.
- Except for recurrent course, there seems to be minimal overlap of ATP with affective disorder.

These research findings could form the basis for any etiological hypothesis that can be invoked for ATP. It is clear that we will have to think of genetic as well as environmental factors to understand its occurrence as well as correlates that have been described above.

Careful examination of triggering factors point to the possibilities that stress (psychological or physiological) together with genetic vulnerability for ATP or for psychotic symptoms are key mechanisms involved in ATP. Association of fever (that was non-specific, self-limiting, without any clinical or lab findings) triggering ATP might point towards the possibility of some infective process such as a viral infection; or towards a hormonal or biochemical abnormality triggered by fever acting as a physiological stress; or fever and possible infection could initiate an autoimmune response affecting the brain functions which could lead to a psychotic breakdown. Association of lower socio-economic status and rural living in ATP patient requires further explanations. Is it to do with poor nutrition or brain damage, which may predispose individuals from lower SES and rural areas, to both fever and psychosis? These hypotheses are only suggestive and need to be tested for validity.

Certain environmental factors occurring early in life have been implicated in the aetiology, course and outcome of schizophrenia some of which apparently overlap with those found in ATP. Schizophrenia in developing country setting has been found to have better outcome. Regarding urban rural differences, urban living has been reported to be an environmental risk for schizophrenia[92,93] and urban risk is cumulative through childhood from birth to 15 years.[94] Prenatal CNS viral infections[95] and drug abuse;[96] antenatal and perinatal CNS damage;[97,98] head trauma in childhood[99] are other factors shown to be associated with schizophrenia. These factors(both genetic and environmental) influence brain function possibly by affecting neuronal cell migration, myelination and pruning during development.

Some of the environmental factors invoked in the aetiology of ATP, such as stress, viral infection, autoimmune response, brain damage, poor nutrition, etc., have also been implicated in the aetiology of schizophrenia with the difference that those occur in later years (adulthood) in ATP and in developmental years in schizophrenia.

There is possibly some overlap of pathophysiological mechanisms between ATP and schizophrenia and these common pathophysiolotical mechanisms may be involved in production of some symptoms of psychosis in ATP or what can be considered a partial syndrome of schizophrenia.

According to Bleuler,[100] schizophrenia is viewed as a single disease entity where the neurophysiological disturbances of indeterminate origin and nature occur leading to dissociative processes which were called as fundamental disturbance of schizophrenia, i.e., autism and associational disturbances, influencing adversely the development of mental capacities in the areas of thoughts, actions and behaviours. Depending upon the individual's adaptive capacity and environmental circumstances, this fundamental process could lead to secondary manifestations such as hallucinations, delusions, social withdrawal and diminished drive.

Some forms of psychoses may have a common genotype. Size and timing of environmental factors influence the genes[101] and neuronal cell migration and pruning during development and they may not be manifested until sexual maturity as in schizophrenia. Similar environmental factors influencing the brain functions during the stage of maturity might produce effects that may produce partial syndromes or variants of schizophrenia and other psychotic conditions. Significant pathophysiological heterogenecity of psychoses has been reported. Psychoses could be seen as the final common pathway caused by several mechanisms. Angst *et al*[102] concluded evidence in favour of a continuum of psychopathological subgroups with a lot of overlap which may differ to a certain extent in respect of course, genetics and response to treatment. By this argument, there is evidence to think that schizophrenia and ATP be considered to lie on a continuum of psychosis. Gerson *et al*[103] further supported the argument with the evidence suggesting that there must be at least gradations of affectivity and schizophrenicity. In future, therefore, we may need to focus on the study of neurobiology of specific psychopathological phenomenon, transcending the discreet disease concepts, e.g., we may study schizophrenic symptoms, affective symptoms or psychotic symptoms. There is also need in future to study people at high risk for psychosis.

In a twin study of genetic relationships between psychotic symptoms in patients diagnosed as schizophrenias, schizoaffective and manic disorders[104] it was found that there was shared familial co-aggregation of psychotic syndrome.

### Sensitive period for brain development and injury

Many cases of schizophrenia are developmental in origin. Most of the models point towards foetal and/or early infancy periods as sensitive periods for the onset of neurodevelopmental insult because these periods are marked by numerous neurological changes.[105] There exists a concept of sensitive periods for brain development and brain injury. Normally, cortical maturation of brain progresses synchronously across all regions during the first 10 years of life. Thereafter, maturational trajectories are independent and uneven. Visuo-auditory, visuo-spatial and somatic regions get maturational peaks by 14-15 and 16 years, respectively. Frontal executive region matures from 17-21 years and pre-frontal cortex matures the last. There are sensitive periods for onset of maturational injury and also for the manifestations of the injury, which means for the onset of the disorder.

In schizophrenia, sensitive periods for neurodevelopmental injury are considered to be:

1. Foetal stage - 3 years: During this stage, important neuroanatomical cortical changes (neuronal proliferation, migration, pruning) take place. Schizophrenia may result from early CNS insult occurring during second trimester and between 1 and 2 years of age.
2. 7-12 years: During this age, myelination is completed.
3. Late puberty: When (a) 40% of neuronal synapses are eliminated, (b) Maturation of temporal and pre-frontal areas occurs and (c) Massive changes in neurotransmitters and body hormones occur.

Propensity to develop schizophrenia or psychotic disorder may result from brain injury occurring early or late in life. Earlier the onslaught of injury, greater is the likelihood of a more severe psychotic illness such as schizophrenia onset of which may be early during childhood or adolescence or later in adulthood, depending upon the severity of brain injury. On the other hand, later the onset of brain injury, as in adulthood, greater is the likelihood of a less severe psychotic illness such as ATP. This supports the theory of continuum of psychosis.

### ATP - a dimensional paradigm

Research on ATP supports the notion that there is:

- Genetic distinctiveness of ATP. Although the findings point towards genetic distinctiveness of ATP, it is hypothesized that ATP may be an environmentally induced psychotic condition superimposed upon an underlying vulnerability to psychosis. What type of psychosis the patient is likely to develop, could be related to:
- The timings of brain insult, e.g., early-life insult may lead more often to schizophrenia and later-life insult may lead to ATP.
- The severity of brain insult where ATP may be associated with less severe insult.

As there are also good outcome schizophrenias, ATP and schizophrenia may lie on a continuum of severity. Liability for psychotic symptoms and the timing of brain insult could serve as the paradigm for distinction.

There may be a common genetic liability to psychosis and further distinction between schizophrenia, MDP and ATP could lie on two dimensions:

- Symptoms dimensions, where symptoms of schizophrenia → schizoaffective psychosis → ATP affective psychosis with psychotic symptoms → affective psychosis without psychotic symptoms lie on a continuum.
- Course dimension, which may vary from chronic deteriorating → recurrent with varying levels of recovery → single episode with full recovery.

Complex interplay between genetic, biological and environmental factors could produce different phenotypic variations recognized in contemporary literature as schizophrenia, MDP or ATP.

In ATP, there may be a genetic liability to psychosis, which may manifest at some point in the course of development, or it may remain latent or not manifest at all. Environmental factors affecting the brain function may trigger this latent pathology or may produce denovo ATP depending upon its severity.

Environmental factors influencing the brain functions during developing the stage might lead to schizophrenia which is a poor-outcome psychosis; and at later stage may lead to either schizophrenia of good outcome or ATP. This hypothesis supports a revival of psychosis as the subject of study transcending the discreet diagnostic categories. However, it also raises certain questions:

- Is there a difference between good-outcome schizophrenia and ATP?
- Is there a threshold of genetic liability that separates ATP from schizophrenia and affective disorder?
- How do environmental factors alter the brain functions leading to psychosis?

Future classification of psychotic disorders may be dimensional rather than categorical. Dimensional concept of psychosis has powerful empirical support in studies showing better prediction of treatment response and outcome by symptom dimensions rather than the categorical diagnoses.[106,107] Psychotic symptoms are seen not just in severely-ill patients in psychiatric hospitals or asylums, but also occur commonly in community samples many of whom do not reach a psychiatric hospital. In a most recent review, Craddock and Owen[30] have summarized problems in using dichotomous classification of psychotic illness and concluded that there is an urgent need to change the current approach. Further, any classification that is only phenomenological- descriptive in nature, as in the DSM system, without a validating biological criteria, will be far from ideal and also subject to arbitrariness in shifting the points of rarity.

Bipolarity and delusional disorders, perhaps, will survive the tests of biological validity,[108] but what kind or level of brain dysfunction will define chronic and acute (my addition) polymorphic psychoses, is hard to guess at this time. The concept of ATP has opened new vistas for further research and theorization even about schizophrenias and affective disorders.
